# Beyond Inverse Dynamics: Methods for Assessment of Individual Muscle Function during Gait

**DOI:** 10.3390/bioengineering11090896

**Published:** 2024-09-06

**Authors:** Stephen J. Piazza

**Affiliations:** Biomechanics Laboratory, Department of Kinesiology, The Pennsylvania State University, University Park, PA 16802, USA; piazza@psu.edu

**Keywords:** gait analysis, muscle function, gait, muscle moment arm, musculoskeletal modeling, gear ratio, induced acceleration analysis

## Abstract

Three-dimensional motion analysis performed in the modern gait analysis laboratory provides a wealth of information about the kinematics and kinetics of human locomotion, but standard gait analysis is largely restricted to joint-level measures. Three-dimensional joint rotations, joint moments, and joint powers tell us a great deal about gait mechanics, but it is often of interest to know about the roles that muscles play. This narrative review surveys work that has been done, largely over the past four decades, to augment standard gait analysis with muscle-level assessments of function. Often, these assessments have incorporated additional technology such as ultrasound imaging, or complex modeling and simulation techniques. The review discusses measurements of muscle moment arm during walking along with assessment of muscle mechanical advantage, muscle–tendon lengths, and the use of induced acceleration analysis to determine muscle roles. In each section of the review, examples are provided of how the auxiliary analyses have been used to gain potentially useful information about normal and pathological human walking. While this work highlights the potential benefits of adding various measures to gait analysis, it is acknowledged that challenges to implementation remain, such as the need for specialized knowledge and the potential for bias introduced by model choices.

## 1. Introduction

Gait analysis typically involves the use of multiple cameras and force platforms to answer research questions about the mechanics of walking and to aid in clinical decision making about the treatment of movement disorders. The modern motion laboratory readily provides a wide array of data that characterize the locomotor mechanics of patients with movement disorders. Body-mounted markers are identified, and their three-dimensional (3D) coordinates are determined with high accuracy hundreds of times per second using multi-camera motion systems, and the synchronized forces and moments applied to the feet by the ground are sampled at even higher rates using instrumented platforms mounted in the floor of the laboratory or integrated into the beds of treadmills. Some of these data are directly useful for the assessment of locomotor function; for example, it may be helpful to visualize how the ground reaction force vector passes relative to lower extremity joints, or to see the forward progression of the center of pressure throughout the stance phase. However, most of the outcome variables from gait analysis are the results of standard post-processing techniques that are incorporated into widely used software packages. Joint angles are computed from the coordinates of markers placed on the body segments using direct kinematics or inverse kinematics methods. Kinematic (motion) data, along with measured ground reaction forces and moments (those applied by the ground to the feet), are used in ‘inverse dynamics’ analyses to estimate the moments and powers produced by muscles and other tissues crossing each joint.

Joint-level estimates of three-dimensional angles, moments, and powers provide information only about the collective actions of muscles, and not about the roles played by individual muscles in producing walking motions. However, knowledge of individual muscle actions is critical because it is the dysfunctions of individual muscles that are addressed by surgeries such as tendon transfers that relocate a tendon’s attachment to bone, tendon lengthenings that alter muscle function, rhizotomies that section nerves to limit overactivity in muscles, or botulinum toxin injections that focally address muscle spasticity. An excessive knee extensor moment may be responsible for a patient’s stiff-knee gait pattern, but the extensor moment must be reduced by treatments that target individual muscles that contribute to the knee moment. Examination of muscle activity may help to elucidate the roles of individual muscles in contributing to abnormal joint moments and powers, and collection of the electrical activity of muscles (electromyography, or EMG) is commonly included in clinical motion analysis protocols. Other noninvasive methods for the investigation of individual muscle function during walking have been developed over the past quarter century, but have mainly been used in research studies.

The purpose of this narrative review is to build on previous reviews (e.g., [1,2]) to survey the available methods for augmenting traditional gait analysis methods through analyses that provide information about the roles of individual muscles in producing healthy and pathological walking motions. Some of the methods described here have been applied to clinical populations and some have not; it is hoped that this review will increase interest in testing the validity of these methods for identifying movement pathology and optimizing treatments. The review concludes with a discussion of the barriers that would need to be overcome in order to add these methods to clinical protocols. While there have been many studies that focus on model-based estimation of individual muscle forces during walking, this topic is beyond the scope of the present review, and interested readers are referred to the excellent systematic review on this subject by Trinler et al. [3]. This was not a systematic review, but an effort was made to find as many relevant papers as possible. The papers cited in this review were identified primarily by searching the National Library of Medicine PubMed database for the terms in the major subheadings of each section followed by cited reference searches of the articles found in PubMed and Google Scholar.

## 2. Muscle Moment Arm

The moment arm of a muscle about a joint spanned by the muscle is the parameter that determines the capacity of a given muscle force for producing a moment about the joint (or joint) it spans. While various methods are available for the estimation of muscle moment arms in vitro [4], options for assessment of muscle moment arm in vivo (and specifically during locomotion) are limited because of the need for the methods to be noninvasive and because the forces carried by muscles and tendons are not easily controlled. Moment arms may be estimated by making kinematic measurements and then computing moment arm as a function of joint angle, as determined from in vitro tests or using a generic computational musculoskeletal model. However, such measures lack consideration of variation across individuals and the potential influence of tendon loading. Manal et al. [5] proposed a “hybrid method” for the determination of the Achilles tendon moment arm that combined 3D video-based motion analysis with ultrasound imaging. Employing an ultrasound probe fitted with tracking markers, the hybrid method allows for the simultaneous 3D location of the tendon and the joint center, from which the moment arm may be computed as the shortest distance from the joint center to the tendon line of action. The same authors used their method to assess moment arm variation over the range of ankle motion and under conditions of contraction or rest in subjects positioned within a dynamometer [6].

### 2.1. Measurement of Muscle Moment Arms during Walking

The hybrid method for muscle moment arm determination was later extended by Rasske et al. [7] to investigate Achilles tendon moment arms during treadmill walking, an approach that revealed variation in moment arm with tendon loading throughout the gait cycle and showed that it was not strictly determined by the joint angle. The location of the ankle joint center was accomplished in the aforementioned studies by placing markers on the malleoli and then locating their midpoint. Using an alternate approach of locating the axis of ankle flexion–extension [8] seems to yield larger Achilles tendon moment arms more consistent in magnitude with estimates obtained from magnetic resonance imaging and accounting for the 3D nature of the moment arm calculation [9]. In addition to the joint center or axis, it may also be important to include consideration of the curvature of the tendon in order to minimize artifact when estimating moment arms during movement [10].

**Figure 1:**
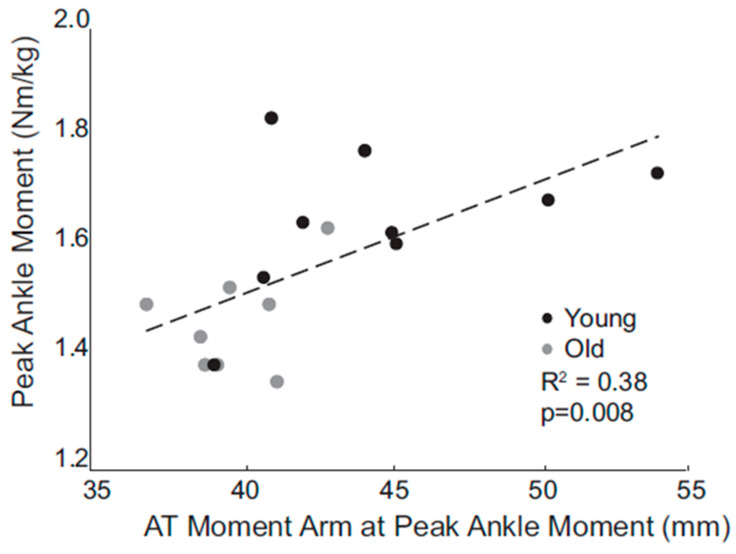
Peak internal plantarflexion moment during the gait cycle plotted versus peak Achilles tendon moment arm, as measured by Rasske and Franz [12] using the hybrid ultrasound and motion analysis technique in young and old individuals. Peak moments as well as peak moment arms were found to be greater in young participants, and an association between moment and moment arm was noted (reprinted from [12] with permission of Elsevier).

Because muscle moment arms depend on muscle–tendon paths and the locations of joint centers, both of which may vary during movement, it may be important to quantify moment arms throughout the gait cycle. Building on their previous experiments involving measurements made during walking [7], Franz et al. [11] further demonstrated the variation of Achilles tendon moment arms with both joint angle, strength of contraction, and the interaction of these factors. This finding underscores the need to interpret the moment arms estimated using conventional musculoskeletal models with caution. The muscle paths and joint kinematics incorporated into these models depend on joint angles alone, and thus lack the capacity for moment arms to vary with muscle contraction [11]. The dependence of the Achilles tendon moment arm on the degree of muscle bulging was cited in another study comparing moment arms measured during the walking of younger and older adults [12]. Older participants were found to have smaller moment arms that corresponded to reductions in plantarflexor moment, suggesting that this is a mechanism underlying age-related decreases in walking speed (Figure 1).

### 2.2. Moment Arm Measurement to Inform Estimation of Force and Stress

Measurement of muscle moment arm during walking may also form the basis for estimates of other parameters of interest that are specific to the individual. Using subject-specific estimates of the Achilles tendon moment arm with measurements of internal ankle joint moment permits the estimation of Achilles tendon force, facilitating the calibration of tensiometers that make tendon stress measurements based on shear-wave elastography [13,14]. With an ultrasound imaging probe placed on the leg, it is then possible to estimate tendon stress during walking (subject to the assumption that the internal moment is due to the triceps surae alone). Achilles tendon forces during walking and running have been estimated with the aid of moment arms measured using the hybrid method in order to investigate potential triggers of the walk-to-run transition that are rooted in tendon dynamics [15].

### 2.3. Muscle Moment Arms in Clinical Populations

Knowledge of moment arms during walking in children with movement disorders could inform decisions about corrective surgeries that are likely to alter moment arms, whether the surgical alterations are intentional or not. While such assessments during walking have not yet been made, there is evidence to suggest that Achilles tendon moment arms in children with cerebral palsy differ from those of typically developing children [16,17]. Differences in bony geometry and muscle size may account for these differences. Further study is needed to address this question, along with investigation of the effects of surgical interventions on moment arms. While some surgeries are explicitly designed to alter function by changing muscle moment arms (e.g., the conversion of rectus femoris from a knee extensor to a flexor via transfer of its tendon), moment arms may be altered even when this is not the primary objective (e.g., changes to the plantarflexor moment arm of the Achilles tendon following hindfoot osteotomies for pes planus).

## 3. Muscle “Gear Ratio”

**Figure 2:**
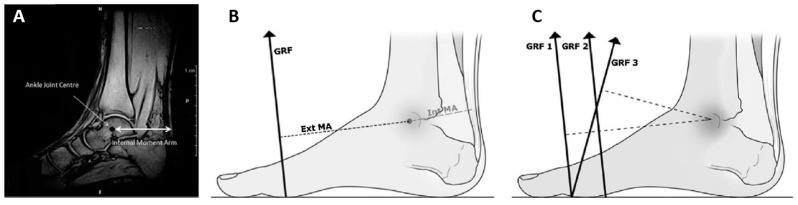
The concept of effective mechanical advantage (EMA) of the triceps surae, as illustrated by Petrovic et al. [20]. The first panel (**A**) shows the ankle joint center and Achilles tendon moment arm superimposed on a magnetic resonance image. The second panel (**B**) shows the lever arm of the ground reaction force (GRF), also about the ankle joint center. The third panel (**C**) illustrates the difference in EMA of the plantarflexors (ratio of tendon moment arm to moment arm of the GRF) that may result from modulating the GRF direction. (reprinted from [20] with permission of Elsevier).

While muscle moment arm is an indicator of the potential for internal joint moment production by an individual muscle, the ability of a muscle to effect locomotion should also take the potential for external forces to produce moments that oppose internal moments into account. The ratio of the output (external force) moment arm about a joint to the input (muscle) moment arm about the same joint has been termed the “gear ratio” [18] because it is similar to the ratio between tooth counts in a pair of adjacent mechanical gears. Alternatively, the inverse of this ratio is the effective mechanical advantage (EMA) [19] of the muscle. Walking with a high gear ratio or a low EMA requires more muscle force to produce the same moment because the muscle is at a leverage disadvantage compared to the external force (typically the ground reaction force), producing the opposing moment (Figure 2).

### 3.1. Foot and Ankle Gearing

So-called “gearing” in the joints of the lower extremities has been linked to locomotor function. Bojsen-Møller [21] suggested that pushoff during walking could be accomplished under “high gear” or “low gear” conditions that could be achieved through the selection of either a transverse metatarsophalangeal axis (high gear) or an oblique one (low gear) that altered the output moment arm. High gear was identified to be ideal for sprinting and low gear was ideal for uphill travel or carrying heavy loads. For normal walking, it was proposed that the foot begins in low gear and transitions to high gear by the end of stance. Carrier et al. [18] similarly noted that the advancement of the center of pressure and (to a lesser extent) changes in the Achilles tendon moment arm at the ankle during the running stance phase cause the plantarflexor gear ratio to vary considerably, effectively “shifting gears” within each step. The authors noted that the gear ratio also dictates plantarflexor shortening velocity, and found shortening velocity to be substantially influenced by gear ratio variation throughout stance. In a study comparing younger and older adults walking at the same speed, it was shown that older adults reduced the demands on the plantarflexor muscles by altering the ankle gear ratio, and differences in the knee gear ratio were noted as well [22].

Modulation of gear ratio seems to be an important mechanism by which the demands on muscles are regulated during locomotion. Differences in EMA between walking and running have been shown to be associated with corresponding differences in joint kinetics that explain variance in the energetic cost of transport between the two gaits [19]. Increasing plantarflexor gear ratio through the addition of stiff insoles into the shoe during walking increased soleus muscle force, decreased its shortening velocity, and increased metabolic energy expenditure [23]. However, the effect of increasing the gear ratio in this way seems to be speed-dependent, with an increased energy cost at low speeds (when a low gear would be more appropriate) and a reduced cost at high walking speeds (when a higher gear is called for) [24]. These results suggest that there are means by which the gear ratio can be set in order to influence plantarflexor muscle function during gait, including the modulation of intrinsic foot stiffness, foot progression angle, and possibly walking velocity and step length.

### 3.2. Abnormal Gearing and “Lever Arm Dysfunction”

The presence of abnormal external and internal moment arms that contribute to problems in the gait of children with cerebral palsy has been termed “lever-arm dysfunction” by Gage [25,26,27], and the influence of joint gearing on cerebral palsy and other movement disorders has been the subject of recent interest. It has been found that children who idiopathically toe-walk have greater plantarflexor EMA (i.e., lower gear ratio) than typically developing children. This difference likely reduces the demands on the plantarflexor muscles during toe walking, perhaps suggesting that caution is needed in planning surgical interventions that might make walking more difficult by normalizing EMA [28]. A similarly higher plantarflexor EMA has been found in persons with diabetes and diabetic neuropathy, revealing a possible mechanism by which the demands on ankle joint muscles may be modulated in these patients [20]. In contrast, patients with Parkinson’s disease have been shown to have lower plantarflexor EMAs than controls, which should increase demands on the muscles [29]. However, when a simultaneous cognitive task was performed during walking, an increase in plantarflexor EMA was observed, suggesting a mechanism by which leverage could be manipulated to reduce effort and improve gait function [29].

## 4. Muscle–Tendon Length

Similar to gear ratio (or EMA), muscle–tendon length is a potentially useful indicator of locomotor function that is not typically included in the reports produced in gait analysis laboratories. Estimation of muscle–tendon lengths requires post-processing of gait kinematics data using a musculoskeletal model. Augmentation of gait analysis in this way has become progressively more accessible to researchers and clinicians over the past 40 years with the advent of specialized software for musculoskeletal modeling, including SIMM [30] and OpenSim [31].

### 4.1. Hamstrings Lengths and Crouch Gait

Interest in understanding how individual muscles contribute to crouch gait in cerebral palsy led to the application of these techniques for this purpose in the 1990s. Crouch gait is a complex movement pattern characterized by excessive hip flexion, knee flexion, and ankle dorsiflexion that has many possible causes, including spasticity and contracture of the hamstrings or hip flexor muscles, or triceps surae that are overly long, perhaps due to previous tendon lengthening. If the hamstrings are determined to be operating at shorter than normal lengths, then hamstrings tendon lengthening surgery becomes a more viable option for normalizing the gait pattern. Hoffinger et al. [32] examined hamstrings length patterns in a series of patients with diplegic cerebral palsy and crouch gait using a computational model driven by measured 3D kinematics data. They found that in the majority of limbs, the hamstrings muscle–tendon lengths were longer during walking than they were at rest, and that length patterns along with EMG indicated that the hamstrings were providing potentially useful hip extension during stance. This finding suggested that hamstrings lengthening could exacerbate hip flexion in some patients with crouch gait. A subsequent study by Delp et al. [33] using a different musculoskeletal model yielded similar results; the majority of patients had hamstrings that were normal length or longer. The psoas, in contrast, was found to be shorter than normal in all the patients with crouch gait, suggesting that hip flexors may be the primary driver of crouch gait in many patients. Thompson et al. [34] also found short hamstrings in only a minority of crouch-walking patients, but extended this analysis by examining the differential response to the application of botulinum toxin among patients with short hamstrings versus adequate-length hamstrings. The former showed a substantial increase in muscle–tendon excursion, while the latter did not. Length and shortening velocity patterns of another biarticular muscle, rectus femoris, were investigated by Jonkers et al. [35] to determine how they relate to levels of spasticity and potentially contribute to a stiff-knee gait pattern.

### 4.2. Muscle–Tendon Lengths of Plantarflexors

**Figure 3:**
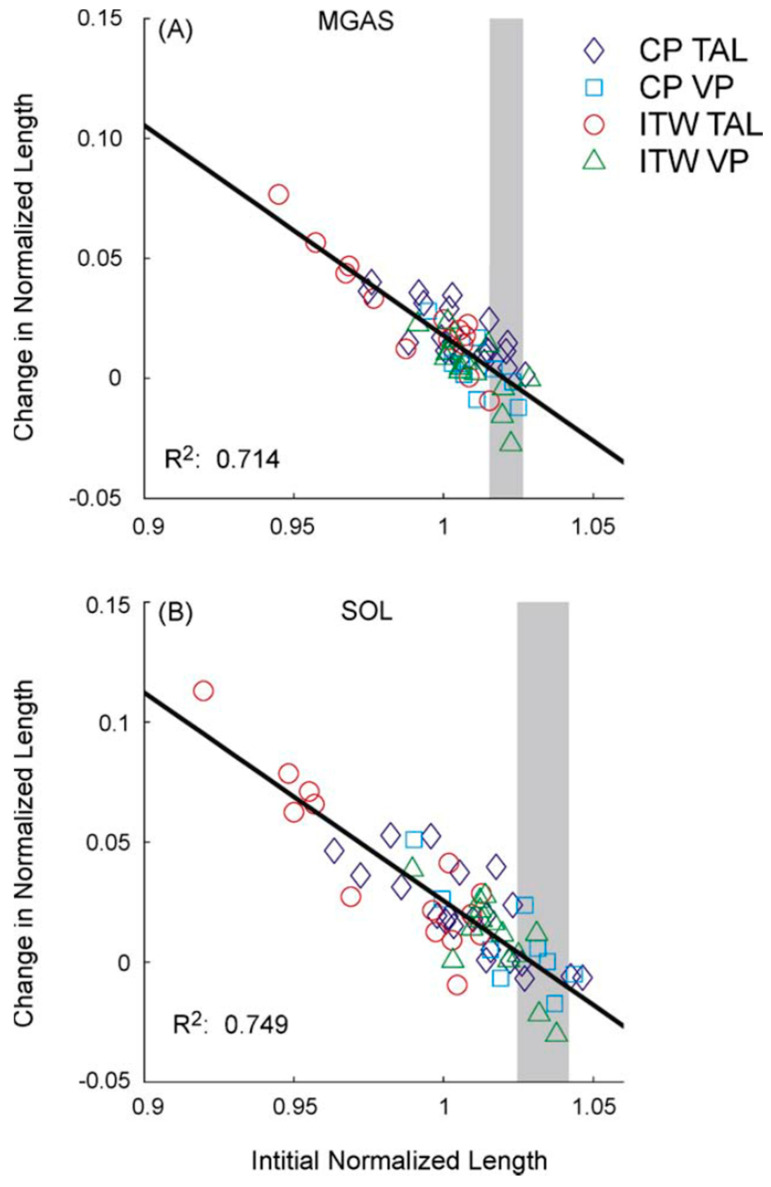
Change in muscle–tendon length for medial gastrocnemius (MGAS; (**A**)) and soleus (SOL; (**B**)) following surgery plotted versus initial length as measured by Jahn et al. [39]. The groups considered included children with cerebral palsy (CP) and idiopathic toe walkers (ITW) who had undergone the Vulpius procedure (VP) or tendo-achilles lengthening (TAL). The gray zones represent the initial muscle–tendon lengths for age-matched controls. The correlations noted by the authors suggested that initial muscle–tendon length, and not diagnosis or another factor, was the major determinant of the amount of the surgical correction. (reprinted from [39] with permission of Elsevier).

Similar techniques have been employed to estimate the muscle–tendon lengths of the triceps surae during walking. Eames et al. [36] used measurements of muscle–tendon paths and joint centers made using magnetic resonance (MR) imaging along with a two-dimensional geometric model to compute patient-specific gastrocnemius lengths from kinematic gait data. After reporting muscle–tendon lengths for typically developing children as well as children with hemiplegic and diplegic cerebral palsy, including some who were pre- and post-surgery, the authors noted the importance of considering biarticular muscle function in surgical planning using model-based processing of gait data. Orendurff et al. [37] used methods that were essentially the same to build two-dimensional models from MR data and compute gastrocnemius and soleus lengths before and after Achilles tendon lengthening surgery, noting improvements in the muscle–tendon lengths that paralleled the changes in the joint angles. Similar investigations using scaled 3D musculoskeletal models to compute muscle–tendon length based on 3D kinematic data have been used to study the effects of tendon lengthening surgery [38,39,40] (Figure 3) and the effect that spasticity has on plantarflexor muscle tendon at different walking speeds [41].

### 4.3. Muscle–Tendon Lengths in Assessment of Injury Risk

Muscle tendon lengths estimated from kinematic data using musculoskeletal models have also been employed to study factors potentially contributing to injury during running [42,43]. Thelen et al. [43] found length patterns in the biceps femoris muscle that were suggestive of heightened risk of injury to that muscle during sprinting, as compared to that of other hamstrings muscles. Riley et al. [42] used this approach to quantify differences in muscle–tendon length patterns between walking and running in multiple hip muscles for the purpose of identifying muscles that would benefit most from stretching prior to running.

### 4.4. Modeling Considerations

Several investigators have conducted investigations of how the muscle–tendon lengths estimated using these methods are affected by modeling choices or by the methods used to collect and process the kinematic data. Schutte et al. [44] made estimates of the hamstrings lengths during gait similar to those described above, but took the further step of recomputing the lengths using a modified model that incorporated femoral anteversion. Scheys et al. [45] noted substantial differences in muscle–tendon lengths estimated for several muscles during gait that depended on whether the musculoskeletal model was built from MR image data or was a scaled version of a generic model. The differences led the authors to advise caution when muscle–tendon lengths are calculated using a scaled version of a generic model of an adult male. Other investigations of methodological aspects of muscle–tendon length estimation have concerned the effects of soft-tissue artifact [46] and the methods used to compute joint angles from marker coordinates [47,48], as well as the choice of the generic musculoskeletal model to be scaled [48].

## 5. Muscle-Induced Acceleration Analysis

In addition to using musculoskeletal modeling to gain insight into musculoskeletal structure and leverage during walking, many investigators have used models to study the dynamics of locomotion, especially with respect to how muscle forces contribute to walking motions. In this section, we review papers that have reported on the accelerations generated by muscles during normal and pathological walking. In these studies, muscle forces may be estimated using inverse dynamics coupled with static optimization or with feedback and feedforward control schemes that produce simulations that reproduce measured gait mechanics [49]. Not included here are studies that use optimization to determine muscle excitations that produce forward-dynamic simulations of novel walking motions from which induced accelerations are computed. Several investigators have computed the accelerations induced by joint moments, but here we limit the discussion to the accelerations induced by specific muscle forces. For a more thorough discussion of muscle-induced acceleration methods and limitations, the reader is referred to the review by Silverman [50].

### 5.1. Methods for Estimation of Induced Accelerations

Accelerations produced by muscle forces are typically computed using a musculoskeletal model that idealizes the body as a chain of rigid segments connected by bilaterally constrained joints. The accelerations of interest may be rotational accelerations at a joint or the acceleration of the whole-body center of mass. To compute the latter, the reaction force arising from a muscle force (and only this force) at the location where the body contacts the ground is computed (e.g., [51]). The components of this reaction force are then divided by the body mass to arrive at the contribution of the muscle force to support (represented by the vertical acceleration) and propulsion (the fore-aft acceleration). The muscle force considered may be a token force (e.g., 1 N) to indicate the potential for muscle force to produce acceleration, or it may be the full force estimated for the muscle from inverse dynamics followed by static optimization to find individual muscle forces.

While the computation of center of mass or joint rotational accelerations arising from muscle forces may be sound, the validity of these accelerations for understanding the roles of muscles has been called into question. Sensitivity studies have shown that muscle-induced accelerations are highly sensitive to the choice of the number of degrees of freedom in the segmented model [52], and that endpoint forces are similarly sensitive to the degree of inter-joint coupling [53]. Some authors have noted this sensitivity as a limitation of their muscle-induced acceleration analysis studies, but there does not yet appear to be a consensus on the conditions under which such analyses are useful for the assessment of muscle function. It is notable that many of the studies cited in this section report similar muscle-induced accelerations during walking, but it is possible that similarity in the acceleration results follows from similarity in the musculoskeletal models used.

### 5.2. Roles of Multi-Articular Muscles

Induced acceleration analysis has been used by several investigators to gain insight into the mechanics of normal walking, often revealing counterintuitive accelerations being produced by biarticular muscles. For example, rectus femoris, which passes anterior to the hip and is thus anatomically classified as a hip flexor muscle, has been shown to produce hip extension acceleration during walking in two studies [54,55], but was found to produce a hip flexion acceleration in a third study [56], although other biarticular muscles were found to produce counterintuitive accelerations in the latter study. It is possible for multi-articular muscles to produce accelerations opposite to their anatomical classifications due to the coupled nature of the linked-segment dynamics; if rectus femoris force produces a reaction force at the knee (the other joint it spans) that passes posterior to the hip, a hip extension acceleration will result. Arnold et al. [57] used an induced acceleration analysis to show that muscles played a greater relative role in controlling knee extension in the late swing phase, perhaps compensating for changes in the velocity-dependent limb dynamics. 

### 5.3. Plantarflexor Contributions to Support and Propulsion

A question addressed by several muscle-induced acceleration studies is that of how the gastrocnemius and soleus contribute to producing the motions of normal walking. Neptune et al. created computer simulations based on experimentally measured data from five individuals and found that both soleus and gastrocnemius contributed to vertical support, but that gastrocnemius had a special role in initiating swing phase motions in the late stance phase [55,58]. These findings were echoed in later studies [54,59] that showed gastrocnemius to play a larger role in forward propulsion than soleus, in that gastrocnemius did more to flex the knee in late stance or produced more anterior acceleration of the center of mass. The accelerations of the trunk produced by individual muscles have also been studied in order to identify their contributions to postural stability during walking [60].

### 5.4. Assessment of Accelerations in Pathological Gait

**Figure 4:**
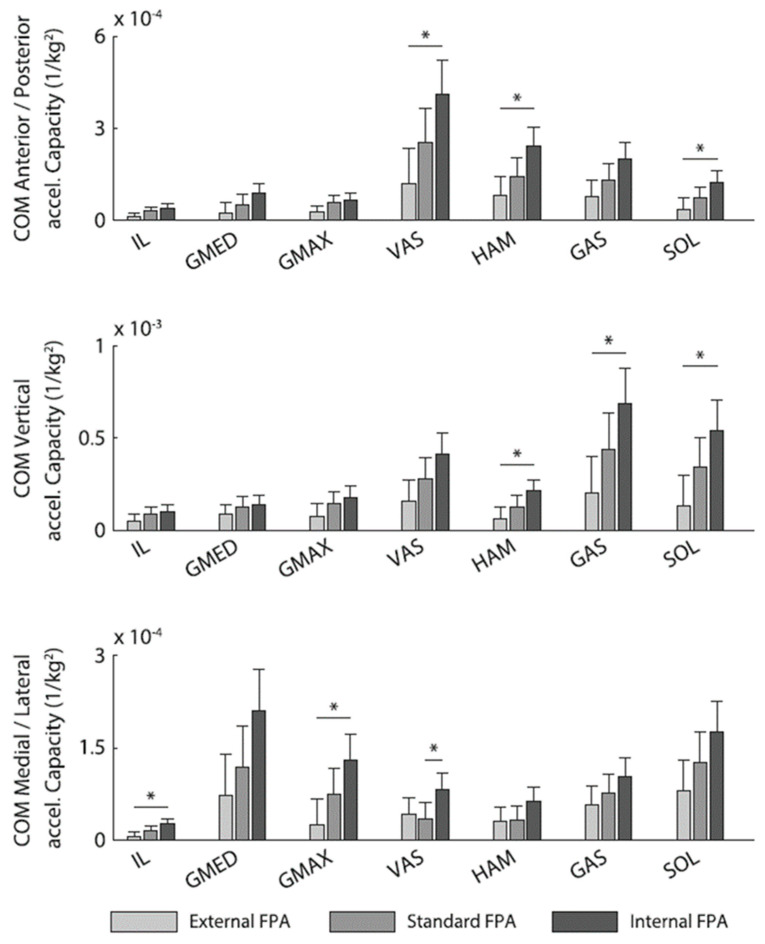
Three-dimensional components of the body’s center of mass acceleration induced by several muscles were estimated using a model-based analysis by Hegarty et al. [62]. Accelerations are presented for three different foot progression angles (FPAs), illustrating the potential for foot rotation to influence the potential for individual muscles to both propel and support the body. Significant differences are denoted with an asterisk (*) (reprinted from [62] with permission of Elsevier).

Similar studies of muscle contributions to center of mass acceleration have been conducted in children with cerebral palsy. Dixon et al. applied these techniques to determine how individual muscles assist in turning [61], finding differences between the roles played by the inside-limb and outside-limb plantarflexors that also varied from the approach to the turn to during the turn itself. Hegarty et al. [62] found that an externally rotated limb posture generally reduced the capacity of major muscle groups for both propulsion/braking and vertical support (Figure 4), a finding that corresponds to the “lever-arm dysfunction” proposed by Gage [24,25,26]. Induced acceleration analysis has been used to study muscle contributions to body accelerations during crouch gait in cerebral palsy [63] as well as in toe-walking executed by individuals without cerebral palsy [64].

Investigators have applied muscle-induced acceleration analysis to a variety of other questions about muscle contributions to walking, including in persons with osteoarthritis [65,66], in persons who have had a stroke [67], and in women who are pregnant [68]. Schloemer et al. [69] found that muscle roles were similar between young and old subjects, but that there were substantial differences in the timing and magnitude of muscle-induced accelerations between young and old. Graham et al. [70] used muscle-induced acceleration analysis to identify which muscles helped to right the body during recovery from a loss of balance that occurred while walking, and which muscles cannot because they are mechanically unable to contribute to fall recovery.

## 6. Conclusions

The studies included in this narrative review are valuable examples of the additional insights that are possible when auxiliary measures and analyses are employed along with conventional gait analysis techniques. Each category of analysis considered moves beyond joint-level mechanics in some respect. Assessment of muscle moment arms during walking is helpful for quantifying the actions of individual muscles subject to the motions and loads that are specific to the gait of individuals. Measurement of gear ratio (or effective mechanical advantage) places muscle moment arms in context with the lever arms of the forces that are opposed by muscle forces on opposite sides of a joint. Knowledge of muscle–tendon length obtained with the use of musculoskeletal models provides critical information about whether surgeries that alter tendon lengths are warranted. Muscle-induced acceleration analysis is intended to indicate the actions of muscles in a manner that takes multibody dynamics into account along with muscle–tendon anatomy.

Nearly all of the studies cited in this review in which additional measurements or computational model-based analysis (or both) are used to gain additional insights from gait analysis represent collaborations between clinicians and engineers or others with advanced technical expertise. The additional expertise needed, including working knowledge of software such as OpenSim [31] or proficiency with ultrasound imaging, is not often found in the staff of a clinical gait analysis laboratory, who already are responsible for a wide array of equipment and techniques such as motion analysis, force platforms, and electromyography. Additional considerations include the time required for these additional analyses and the potential for a lack of trust in the models and measures [48]. Kainz and Schwartz [48] studied the effects of simplifying the implementation of musculoskeletal modeling methods for augmenting clinical gait analysis, and found that the computation of muscle–tendon lengths could be accomplished using joint angles, eliminating the need for model scaling and inverse kinematics performed in musculoskeletal modeling software such as OpenSim.

More attention is needed to the effects of musculoskeletal model choice in model-based assessments of parameters like muscle–tendon lengths. Many of the studies cited here have used the same musculoskeletal model that has been scaled to match the size of the subject. Research on the effects of model choice, such as the investigation of model complexity conducted by Sohn et al. [71], can help to address this question. If simpler models with fewer degrees of freedom and fewer muscles can be shown to provide similar insights as those derived from more complex models, it will facilitate the interpretation of modeling results for the purpose of clinical decision-making.
